# An Experiment in Personalized Shopping for Optimal Health, with Integration of Nutrigenetics and Gut Microbiome Information

**DOI:** 10.3390/nu18101528

**Published:** 2026-05-12

**Authors:** Veronica Fernandes, Magalí Pezzarini, Yamile Márquez, Cláudia S. Marques, Facundo Ballesteros, Arnau Carmona, Priscila M. S. Delgado, Jéssica Fernández, Luciano Heitt, Darmit M. Kumar, Ana C. Magalhães, Nahuel Rosas, Keyvan Torabi, Marina Riera, Jean Pierre Lannou, Luisa Pereira

**Affiliations:** 1 i3S—Instituto de Investigação e Inovação em Saúde, Universidade do Porto, R. Alfredo Allen 208, 4200-135 Porto, Portugal; 2 GUNDO Health and Food SL, C. Gordoniz 53, Entreplanta Izquierda, 48002 Bilbao, Spain; 3 ADN Institut, Institut d’Assessorament i Diagnòstic Genètic SL, Av. de Cerdanyola, 08172 Sant Cugat del Vallès, Spain

**Keywords:** personalized nutrition, multi-omics integration, digital health, e-commerce nutrition, food choice behavior

## Abstract

**Background**: An individual’s health status is determined by the interaction of genetic, environmental, social, and lifestyle variables. Nutrition plays a fundamental role in disease prevention, a notion widespread among the informed public, which is keen on participating in initiatives for personalized recommendations informed by advanced biological data. **Objectives**: To evaluate whether a personalized nutrition service can produce measurable changes in nutritional behavior and biological outcomes, we established the GENIE digital platform. This platform delivers personalized online food shopping and recipe recommendation informed by integrated data from (i) biochemical blood markers, (ii) nutrigenetic profiles, (iii) gut microbiota composition, and (iv) consumer preferences. **Methods**: We conducted a single-arm study in a Spanish cohort that used a specific online retailer for food shopping, totaling 1177 participants. Group 1 (*n* = 620) had recommendations based only on the biochemical blood test; Group 2 (*n* = 357) included the nutrigenetic test; and Group 3 had the gut microbiome test (first batch, *n* = 200; second batch, *n* = 97). After one month of informed, tailored dietary advice, a quantitative evaluation of the experience was conducted. **Results**: The GENIE platform led to strong engagement (mean session time 7.07 min; +154% e-commerce use), with 71% of participants following at least part of the recommendations. This was associated with an increase in microbiome diversity in about 70% of participants, after just one month of guided recommendations. **Conclusions**: The GENIE platform represents a pragmatic model for translating nutrigenetic and microbiome data into actionable dietary recommendations, bridging the gap between scientific evidence and consumer behavior.

## 1. Introduction

Human health is determined by the interaction of dynamic and complex intrinsic (e.g., genetic background, age, or gender) and extrinsic (such as diet, physical activity, and environment) factors [1]. Research over the last two decades has intensively focused on the genetic and environmental interactions underlying complex diseases that constitute contemporary health challenges in high/middle socio-demographic index countries, such as type 2 diabetes, obesity, hypertension, cardiovascular disease, and late-onset neurodegenerative disorders [2,3]. The development of high-throughput genomic technologies such as microarrays and next-generation sequencing has revolutionized biomedical research and clinical practice, allowing a personalized medicine approach [4,5]. These tools allow the characterization of the individual’s genomic background, providing insights into genetic predisposition to diseases, supporting diagnostic stratification, and more targeted and effective therapeutic strategies [6]. One of the most promising aspects of personalized medicine is disease prevention, in which evaluation of the genetic risk for complex diseases can guide tailored interventions and promote behavior changes in order to reduce the chances of disease manifestation [6,7].

Diet, together with physical exercise, is the main environmental factor to prioritize for intervention to improve health outcomes. As such, nutrigenetic and nutrigenomic testing based on individual genetic variation affecting nutrient response, and evaluating nutrient-driven changes in gene expression, along with microbiome profiling, particularly of the gut microbiota, have been widely explored [8,9]. Indeed, controlled feeding experiments proved that even small alterations in macronutrient composition can induce measurable changes in microbiome structure within just a few days [1,9], and individual genetic variants also shape dietary responses [8]. Analysis of the gut microbiome is usually performed on stool samples, where microbial DNA is extracted and sequenced to characterize community composition. The traditional method, 16S rRNA gene sequencing, allows taxonomic profiling of bacterial communities, while shotgun metagenomic sequencing provides higher-resolution data on microbial species and their functional and metabolic potential [8,10], allowing linking microbiome composition and diversity with dietary patterns, obesity, insulin resistance, and other cardiometabolic features [11]. In parallel, nutrigenetic testing is typically carried out using DNA collected from saliva, buccal swabs, or blood, followed by genotyping through single-nucleotide polymorphism (SNP) arrays or next-generation sequencing. The resulting data are used to identify genetic variants in genes that have an impact on the individual’s health in terms of absorption of vitamins and minerals, food intolerances, response to diet, eating habits, predisposition to metabolic pathologies, or response to exercise, among others [12]. For instance, genetic variants of the fat-mass and obesity-associated (*FTO*) gene have been correlated with high body mass index (BMI), increased adiposity, predisposition to obesity, metabolic syndrome, and type 2 diabetes mellitus [13,14], with evidence that physical activity can modify the effects of this genetic predisposition [15]. Similarly, carriers of the APOE4 allele exhibit greater LDL-cholesterol in response to saturated fat intake compared with non-carriers, proving how genotype can alter diet–lipid interactions [16,17], which can then be translated into personalized dietary recommendations [12]. Together, microbiome and nutrigenetic assays provide complementary insights into host–diet interactions, offering the potential to inform evidence-based personalized nutrition (PN) strategies.

A growing body of evidence from diverse study designs—including large prospective cohorts, machine-learning prediction models, and randomized dietary interventions—has demonstrated the potential of multi-omics data to inform PN. Studies from large, well-characterized cohorts indicate that individuals can exhibit markedly different postprandial metabolic responses to the same foods, and that these variations can be partly predictable using personal features such as microbiome composition, dietary habits, clinical parameters, and lifestyle factors [18,19]. Algorithm-informed PN has evolved from proof-of-concept to randomized interventions, including a personalized nutrition project using a machine-learning algorithm integrating clinical, lifestyle, and microbiome data that accurately predicted individual postprandial glycemic responses [20]. The algorithm-based diets designed by this model significantly reduced postprandial glycemia and induced consistent changes in gut microbiota composition [20]. In addition, findings from the Personalised Responses to Dietary Composition (PREDICT) program showed inter-individual variability in glycemic and lipemic responses, and indicated that integrating personal, dietary, and microbiome data provides stronger predictive power than nutrient-based information alone [18]. At the dietary–microbiome interface, a randomized dietary trial (Fermented and Fiber-rich Food—FeFiFo Study) indicated that fermented-food-rich diets can increase gut microbiome diversity and decrease inflammatory markers, suggesting that targeted diets can significantly shape host–microbe interactions [21]. These results support PN approaches that transform individual risk profiles into specific food selections, recipes, meal planning, and shopping guidance. Recently, an 18-week app-based PN program that integrated postprandial responses and microbiome features reported improvements in outcomes such as body weight, waist circumference, HbA1c, diet quality, and microbiome diversity, particularly among participants with high adherence, highlighting the importance of algorithm design and engagement for intervention success [22]. Together with microbiome and digital-driven interventions, evidence from nutrigenetic approaches further demonstrates how incorporating genomic information can increase the effectiveness of personalized nutrition strategies. Indeed, the Food4Me European randomized controlled trial provided further evidence that genotype-informed dietary advice can influence outcomes, including in participants who carry the FTO risk alleles, who showed significant 6-month reductions in body weight and waist circumference compared with non-carriers [23]. More recently, the NOW trial demonstrated that the provision of nutrigenetically based information derived from 12 obesity and metabolism-related genetic variants, set within a lifestyle program, resulted in a significant reduction in body fat percentage compared with standard recommendations [24], reinforcing the translational potential of genomics-guided nutrition.

Nutrition plays a fundamental role in disease prevention, a belief reflected by consumers who are motivated to adopt PN services that provide dietary recommendations tailored to their individual needs and preferences [25]. In recent years, direct-to-consumer (DTC) PN platforms have expanded rapidly, highlighting the strong demand for individualized dietary advice, but also the challenges of their scientific validation and practical implementation. Several companies (e.g., ref. [26]) offer services that combine elements of genetic testing, microbiome analysis, and digital coaching, but their outputs are typically delivered as static reports with limited follow-up or by app notifications, lacking behavioral follow-up, poorly integrated within the food-shopping environment, and with little evidence of either systematic usability validation or broader user-centered evaluation. Importantly, implementing ethical requirements in nutrigenomic and nutrigenetic testing is critical for consumer safety. Under the EU’s General Data Protection Regulation (GDPR), platforms that collect health-related data—including genetic or microbiome information—from EU residents must comply with strict requirements regarding personal data processing, consent, data subject rights, and secure storage [27]. The PREVENTOMICS mFood platform [28] is an example of a multi-omics personalized nutrition system aimed at preventing diet-related diseases, built within ethical and legal frameworks, including compliance with GDPR.

Our project was designed to evaluate a supermarket-integrated PN tool—the GENIE (Genomic Evaluation and Nutritional Integration Experience) platform. This tool brings together nutrigenetic information, gut microbiome profiling, routine clinical biomarkers, and lifestyle/preferences to create individualized profiles that would translate into practical outputs such as personalized dietary advice, product recommendations, shopping lists, and recipe suggestions. The main objective was to determine whether the use of this tool is associated with measurable changes in nutritional behavior and biological outcomes. In addition, the project also aimed to (i) demonstrate how personalized nutritional and groceries recommendations may be associated with an improvement in the patient’s wellbeing by comparing the composition of the gut microbiota before and after the personalized shopping experience; (ii) evaluate users’ understanding of genetic and microbiome risk displays using specific comprehension surveys. This study addressed a key translational gap by implementing multi-omics PN directly into routine shopping decisions and evaluating both biological markers and user experience within a unique pragmatic framework.

## 2. Materials and Methods

### 2.1. Selection of an Online Food Retailer Partner

The GENIE project established a collaboration with the retailer Ametller Origen, which holds a strong position in the online food sales market in Catalonia, Spain. This strong online position made it ideal to leverage the Ametller Origen e-commerce platform and users’ loyalty accounts. However, the GENIE digital platform is easily adapted to connect with other retailers.

### 2.2. Establishing the GENIE Digital Platform

The GENIE modular, user-centered digital platform was designed to translate complex health data into personalized nutritional guidance. The platform integrates health diagnostics, enriched product data, and supermarket systems to deliver individualized recommendations, which are directly applicable to daily grocery purchases. The platform is organized into three modules.

#### 2.2.1. Enriched Product Data

A central component of the GENIE platform is the transformation of the supermarket product assortment into a structured, health-actionable database. We developed and applied a proprietary nutritional enrichment pipeline to more than 3800 products, extracting and evaluating an average of 4000 attributes per item. These attributes encompassed macronutrient and micronutrient composition, presence and classification of additives and allergens, ingredient categories, processing levels, and 46 predefined nutritional profiles (e.g., high-fiber, low-sugar, anti-inflammatory). The enrichment process employed 11 internal analytical layers that systematically tagged and scored each product according to diverse health-related criteria. This enabled classification of items into suitable, neutral, or non-recommended categories, depending on individual health data and nutritional objectives. Each product entry also incorporated extended information, including ingredient explanations, processing-level justifications, allergen and additive insights, and product certifications (e.g., organic, gluten-free).

#### 2.2.2. Data Center (User Vault)

The Data Center, or User Vault, functions as the central repository for secure storage and management of health-related data, supporting structured uploads, automated interpretation, and generation of personalized insights. It was designed to accommodate multiple diagnostic modalities:

Blood and urine tests: Users can upload laboratory blood results directly into the platform. Reports are parsed to extract relevant biomarkers, which are classified as optimal, borderline, or critical. Urine analysis was also incorporated using the same approach. The biomarker engine currently supports over 100 indicators, mapping results to evidence-based nutritional strategies. Sequential tests can be uploaded, and the historical tracking enables monitoring of progress and early detection of deviations.

Microbiota and nutrigenetic tests: These modalities are fully integrated via APIs. Users can follow kit status (ordered, in transit, under analysis) and access simplified summaries, full scientific reports, and personalized nutritional recommendations. Results are enriched with visual analytics, including microbiota diversity indices, genetic predisposition heatmaps, and correlations between genotype and nutrient metabolism.

The vault also incorporates alert systems for critical parameters, notifications to update outdated results, and recommendations to seek professional consultation when required. Alert thresholds can be customized by users or their nutritionists. The personalization engine adapts dynamically to the available data, ensuring that recommendations are generated even when some biomarkers are missing.

The system is built on a GDPR-compliant architecture with advanced encryption, multi-factor authentication, and auditable consent management to ensure privacy and security. Users retain full control over their data, with the ability to edit, delete, download, and manage consent at any time. Consent is granular, allowing opt-in or opt-out for specific applications (e.g., research, AI model training, or personalization services). All data interactions are logged, and users can review a complete history of data usage within the platform.

#### 2.2.3. Personalized User Experience and Supermarket Integration

The personalization engine provides individualized nutritional guidance by combining the enriched product database with each user’s health profile. Recommendations are generated through multiple analytical layers, including compatibility assessment, nutrient gap evaluation, and preference-based modeling, to deliver highly personalized nutritional guidance (Supplementary Figure S1 in File S1). By integrating these layers, the system identifies the most suitable recommendations for each individual, aimed at optimizing health outcomes while aligning with user relevance. This approach enhances overall effectiveness by balancing what is nutritionally ideal with what is practical and sustainable in the context of each person’s daily life, ultimately supporting long-term adherence and impact.

Multi-omics and behavioral integration: Personalized nutritional recommendations and diet plans are generated through the integration of multi-omics and behavioral data, including nutrigenetics, gut microbiota composition, blood biomarkers, and lifestyle questionnaires. Each layer contributes unique insights that are computationally integrated to derive evidence-based, personalized nutrition strategies that align genetic predispositions with real-time metabolic and lifestyle information, ultimately improving adherence and health outcomes.

Meal plan generator: A core feature is the dynamic meal-planning segment, which incorporates individual health goals, dietary preferences, culinary habits, budget constraints, and cooking time availability. Users can define plan duration, select the number of meals per day, and exclude specific ingredients. Outputs are displayed in calendar format with daily details, linked to step-by-step recipes and supermarket products. Plans can be modified, regenerated, or exported as PDFs. The generator uses a nutrient-balancing approach to align recommendations with established dietary intake ranges, while also considering cultural, seasonal, and economic considerations. It incorporates real-time product availability from Ametller Origen’s stock to minimize the need for substitutions due to out-of-stock items.

Product-level personalization: Beyond meal planning, users can browse the full supermarket assortment through a personalized health filter. Products are classified as suitable or non-suitable, accompanied by clear justifications and extended details (ingredients, additives, allergens, nutritional profiles, processing levels, and certifications). Recommendations adapt in real time to updates from the User Vault, ensuring continuous personalization.

Smart shopping and omnichannel integration: All actions contribute to a personalized shopping list, which can be exported, connected with the e-commerce platform, and linked to the user’s loyalty account. The architecture is designed to be scalable, supporting deployment with additional retail partners using the same personalization core.

### 2.3. Application Programming Interfaces

Several integrations were developed in the GENIE platform with the retail system, via Application Programming Interfaces that conform to the design principles of the Representational State Transfer architectural style (REST API). A product catalog API enabled regular synchronization of the assortment, including information such as ingredients, nutritional values, origin, price, and nutritional profile pictograms. This data was subsequently enriched within the GENIE platform—another API integration with the retail loyalty program validated and linked user identities between the involved partners. A further API allowed personalized shopping lists to be transferred to the retail’s digital ecosystem, enabling users to manage these lists directly via their app or e-commerce platform. Finally, through Secure File Exchange, the real purchase histories of participants were obtained, allowing the authors to assess behavioral changes before and during the intervention. The orchestration was secured in cloud microservices (Google Cloud).

### 2.4. Participants

A pre-launch phase (Phase 1) was established with Ametller Origen employees (*n* = 96) to gain experience with the entire process. The proper experimental phase (Phase 2) recruited participants through a digital campaign distributed via the Ametller Origen daily newsletter to members of the Ametller Club. A brief announcement in the Sunday edition introduced the project and directed recipients to a dedicated landing page, which contained study information and a registration form. Eligibility criteria included residence in the metropolitan area of Barcelona, active membership in the Ametller Club, and completion of a personalized nutritional profile within the platform. Enrollment was determined on a first-come basis among individuals fulfilling all criteria. Overall, 120,956 unique users opened the newsletter, 5308 accessed the landing page, and 3199 registered within 48 h. From these, 1000 participants were enrolled, corresponding to the maximum number allowed by the study design (constraints dictated by budget and a 1-year time frame of the EU-funding project). A small number of additional participants were incorporated in phase 2 to expedite the collection of the biological samples, resulting in a total of 1177 participants.

All participants provided written informed consent prior to sample collection. The consent form, prepared and validated by the Data Protection Officer of ADN Institut (a certified healthcare center in Spain), detailed the scope and purpose of the genetic and microbiota testing, as well as the legal basis for data processing. In particular, participants expressly authorized the collection and analysis of biological samples and agreed to the processing of their personal, genetic, and health-related data in compliance with the EU GDPR (Regulation (EU) 2016/679) and the Spanish Organic Law 3/2018 on the Protection of Personal Data and guarantee of digital rights (LOPDGDD). Importantly, the consent form included a clause informing participants that their anonymized data could be used for scientific research purposes in the fields of nutrition and health. Participants were also informed of their rights to withdraw consent at any time and to exercise access, rectification, deletion, or opposition over their data. The data were stored on secure servers and processed with appropriate technical and organizational safeguards to ensure confidentiality and data protection.

### 2.5. Questionnaires

Participants completed a series of structured questionnaires (File S2) designed to collect detailed information on dietary habits, lifestyle, clinical background, and behavioral traits. All questionnaires were self-administered online, through the GENIE web platform, via a secure interface. Mandatory fields and logic checks were implemented to ensure data completeness and consistency. Data collection complied with GDPR regulations, and explicit informed consent was obtained at each stage. Three levels of questionnaires were implemented:

Baseline Nutritional Profile (Questionnaire 1—Personalized Nutrition Data Intake Form): Administered to all participants at registration. Items covered dietary preferences and restrictions (e.g., vegetarianism, lactose intolerance), frequency of food consumption, cooking habits, self-reported weight and height, physical activity, sleep patterns, and general wellness indicators. Data from this survey were used to generate an initial personalized nutritional plan and to confirm compatibility with the proposed interventions.

Advanced Screening Questionnaire (Questionnaire 2—Clinical history, psychological and behavioral data related to eating): Administered to participants fulfilling eligibility criteria and opting for deeper personalization. This survey collected detailed clinical history (e.g., chronic conditions, digestive disorders), psychological and behavioral traits related to eating (e.g., emotional eating, binge tendencies), vitamin and mineral intake sources, adherence to dietary patterns (e.g., Mediterranean diet), and previous responses to dietary interventions.

Post-intervention Feedback Survey (Questionnaire 3—GENIE Experience Feedback): Offered after participants received personalized reports. This optional survey assessed comprehension of results, perceived usefulness of recommendations, satisfaction with the process, and anticipated impact on future dietary behaviors. Data was used to evaluate user experience and the perceived value of the GENIE solution.

### 2.6. Nutrigenetic Analyses and Reporting

Saliva samples were self-collected by the participants using a saliva collection kit (REAL Saliva DNA Sample Collection Kit, Durviz, Valencia, Spain). DNA was extracted from the samples following a protocol based on magnetic bead purification (MagicPure^®^ Genomic DNA Kit, TransGen Biotech Co., Beijing, China) and quantified using a fluorometric method (Qubit 4.0, ThermoFisher Scientific, Waltham, MA, USA). The 96-well array plate version of the Axiom™ Precision Medicine Diversity Array (ThermoFisher Scientific) was used to run all the samples on the GeneTitan instrument (Thermo Fisher Scientific). The standard manufacturer’s protocol was followed for all steps of the workflow. Genotype calling was performed in the Axiom Analysis Suite v4.0.1. Only samples with data quality control (DQC) > 0.82 were retained for nutrigenetic analyses. DNA from the samples with low DQC values was reanalyzed on a new plate. If a sample had a low DQC in a second trial, a new DNA sample was obtained and analyzed de novo. A total of 357 samples were processed, and genotype data were obtained for each sample in a variant call format (VCF) file.

The nutrigenetics report comprises a total of 300 polymorphisms in 160 different genes distributed in seven main categories (Vitamins and minerals, Food intolerance, Dietary habits, Response to diet, Metabolic diseases, Response to physical exercise, and Methylation profile) and 39 subcategories covering different nutritional features. Axiom probes targeting the 300 polymorphisms in the nutrigenetics report were retrieved from the 357 VCF files. If the genotype calling of a given genetic variant in one or more samples failed, a manual inspection of the probe cluster was performed. Only those samples that were located inside a genotype cluster were assigned to the given genotype; otherwise, they were kept as non-informative. For each of the 39 subcategories of the nutrigenetics report, a risk label (no risk, low risk, medium risk, moderate risk, high risk, favorable, unfavorable, neutral, and non-informative) was obtained considering the combination and the presence of risk, protective, and neutral alleles in a given subcategory for all 357 samples. In addition, a nutritional/lifestyle recommendation was elaborated for each type of risk label in each subcategory. The final automatic nutrigenetics report was implemented in PyFPDF (https://pypi.org/project/fpdf/, accessed on 1 October 2024) using the risk labels and nutritional/lifestyle recommendations obtained from the genotype analyses for each individual.

### 2.7. Gut Microbiome Analyses and Reporting

A subgroup of the participants in the nutrigenetics test (*n* = 200) also provided a stool sample for gut microbiome analysis. All of them were further invited to provide a second sample around one month after receiving the first microbiome report, but only 97 individuals did so. The samples were collected by the users themselves using a stool collection and preservation kit (Stool Nucleic Acid Collection and Preservation Tube Dx; Norgen Biotek Corp., Thorold, ON, Canada). DNA extraction for each sample was based on magnetic bead purification and performed following the manufacturer’s instructions (MagicPure^®^ Soil and Stool Genomic DNA Kit; TransGen Biotech Co., Beijing, China) and quantified by fluorometry (Qubit 4.0, Thermo Fisher Scientific). The variable regions V3-V4 of the 16S rRNA bacterial gene were amplified using primers 515F (5′-GTGCCAGCMGCCGCGGTAA-3′) and 806R (5′-GGACTACHVGGGTWTCTAAT-3′). Amplified PCR products were verified by electrophoresis on a 2% agarose gel, and then pooled in equimolar ratios for magnetic bead purification, end-repaired, A-tailed, and further ligated with Illumina adapters. Sequencing libraries were generated and quantified with Qubit 4.0 (ThermoFisher Scientific) and real-time PCR, while a 2100 Bioanalyzer Instrument (Agilent Technologies, Santa Clara, CA, USA) was used for size distribution detection. The sequencing was performed on the Illumina NovaSeq 6000 platform (Illumina, San Diego, CA, USA), generating 250 bp paired-end reads. Paired-end reads were assigned to samples based on their unique barcodes and truncated by removing the barcode and primer sequences. Paired-end reads were merged using FLASH [29]. Quality filtering of the raw reads was performed using fastp (v0.23.1) to generate high-quality clean tags [30]. Effective tags were obtained by removing chimeric sequences with the vsearch package [31] after comparing tags against the reference database SILVA for 16S/18S rRNA genes (https://www.arb-silva.de/, accessed on 1 February 2025). Denoising of the effective tags to obtain initial Amplicon Sequence Variants (ASVs) was carried out using the Divisive Amplicon Denoising Algorithm 2 (DADA2) [32] in the QIIME 2 software (version QIIME 2 2022.2). Species annotation was performed using the Silva Database [33] within QIIME 2 software. The absolute abundances were normalized using a standard of sequence number corresponding to the sample with the fewest sequences.

For the individual report, 32 features of the individual gut microbiome diversity were compared with published data from healthy Spanish/other European data (genera relative abundance data extracted from [34]). The reference data included microbiome profiles inferred from sequencing the same V3-V4 16s rRNA regions (ASV analysis) for 194 individuals (56% from Spain and 44% from other European populations). Principal component analysis allowed us to check for the absence of batch effects and to identify outliers that were removed. These features covered a broad spectrum of gut microbial activities, including nutrient degradation (proteins, lipids, carbohydrates, mucins), metabolite production (short-chain fatty acids, vitamins, methane, indoles), and xenobiotic metabolism, and the information was collected through a careful review of the literature. As an example, for the “Methane” feature, we included the genera *Methanosphaera*, *Methanobrevibacter*, *Methanobacterium*, *Methanosarcina*, *Methanococcus,* and *Methanospirillum*. The features also included information on overall phyla and genera profiles, diversity (Shannon index), and presence of pathogens (*Clostridioides*, *Enterococcus*, *Klebsiella*, *Morganella*, *Salmonella*, and *Shigella*). The reference database was used to obtain a unimodal distribution for each feature (except for phyla and genera profiles, and presence of pathogens; the distribution was not normal for the methane-producing bacteria), against which the specific individual value was compared, and categorized as neutral (reference-like values), moderately dysregulated (in the intervals [2.5–25%] or [75–97.5%], for beneficial and prejudicial features, respectively), and highly dysregulated (in the intervals [0–2.5%] and [97.5–100%], for beneficial and prejudicial features, respectively).

RStudio software (Posit, Boston, MA, USA; version 2025.05.1+513) was used for the overall statistical analysis of the microbiome data for the 200 individuals in the first batch. Clustering of sample microbiome categories was performed within a heatmap framework constructed from scaled feature values. Hierarchical clustering of both rows (samples) and columns (categories) was carried out using Ward’s minimum variance method (Ward.D2) applied to a Euclidean distance matrix. The optimal method and number of clusters were chosen after analysis and validation of Silhouette, Calinski–Harabasz, and Dunn indices. To test if the microbiome profiles differ in participants between the microbiome batches, the non-parametric Bray–Curtis distances were calculated in QIIME to quantify differences in community composition between before/after batches, based on abundance data. A PERMANOVA test was then performed to assess whether overall community composition differed significantly between groups. To account for the sensitivity of PERMANOVA to differences in within-group variability, a PERMDISP test was conducted to evaluate the homogeneity of dispersion among groups. The non-parametric Wilcoxon rank-sum test was also used to identify individual microbial functions that contributed to the observed differences between the clusters. *p*-values were corrected for multiple testing using the Benjamini–Hochberg false discovery rate (FDR) method, with adjusted *p* < 0.05 considered statistically significant. The 95% confidence intervals (95% CI) for the proportions of neutral, moderately dysregulated, and highly dysregulated were also calculated using the modified Wald method [35].

## 3. Results

### 3.1. The Establishment of the GENIE Digital Platform

**Figure 1:**
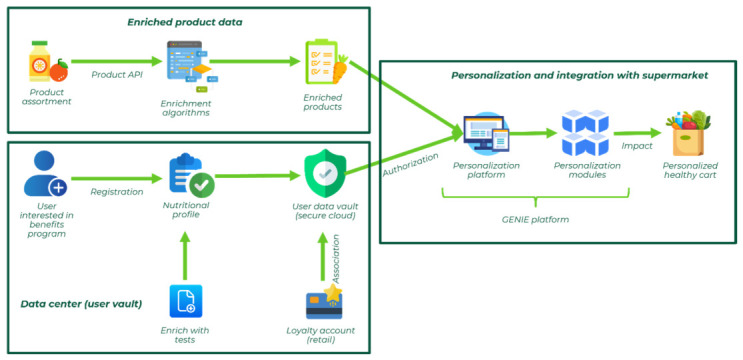
Architecture of the GENIE digital platform, illustrating the integration of the three modules: Enriched product data; Data center (user vault); and Personalization and integration with the supermarket.

An important output of this project was to design an efficient and user-friendly online platform. The most important challenge was to integrate complex data, including health diagnostics, nutrigenetics, and gut microbiome tests, questionnaires, enriched product data, and supermarket systems. A modular design (Figure 1; Supplementary Figures S2–S4 in File S1 display print-screens of the online platform) was implemented for greater operational efficiency:

1. Enriched product data. A nutritional enrichment layer applied to the entire supermarket assortment, converting each product into a structured dataset comprising more than 4000 parameters mapped against 46 predefined nutritional profiles. This framework not only supported the personalization engine but also improved transparency and user understanding of food quality and composition.
2. Data Center (User Vault). A secure (GDPR-compliant architecture), privacy-compliant data module enabling users to upload, manage, and visualize health-related information, including blood, urine, nutrigenetic and microbiota results. Each layer contributes unique insights that, when combined, provide a comprehensive picture of individual nutritional needs. For example, nutrigenetic analysis can reveal polymorphisms affecting folate metabolism, guiding increased dietary folate intake; microbiota profiling can highlight reduced fiber-fermenting bacteria, suggesting higher prebiotic consumption; blood biomarkers such as elevated homocysteine or abnormal lipid profiles can point to targeted adjustments in micronutrient or fat intake; and questionnaires capture patterns such as frequent meal skipping or food preferences, which ensure that recommendations remain practical and individually aligned. Automated interpretation generates global nutritional insights and personalized alerts, which materialize as a dynamic meal planning segment and direct personalized shopping list connected with the e-commerce platform.
3. Personalized user experience and supermarket integration. By combining individual health, nutrigenetic, and gut microbiome parameters with the enriched product database, the platform produces tailored recommendations, meal plans, and product lists, integrated with the supermarket’s e-commerce, loyalty, and in-store systems.

This architectural integration allowed a feasible operationalization of omics-based health data into real-world nutritional interventions. As a scalable pathway for personalized nutrition within retail environments, GENIE makes it easier for users to engage with personalized recommendations when buying food or following suggested daily recipes.

### 3.2. Characterization of the Cohort

The registration questionnaires allowed the characterization of the cohort (Supplementary Table S1 in File S3). Of the 1177 participants, 18% were men and 82% were women. The majority of participants were aged 31–50 years (60%), followed by >50 years (31%) and 18–30 years (9%).

Regarding lifestyle, 80.2% reported practicing physical activity, with a mean of 3.9 sessions per week and an average duration of 129.5 min per week. Most participants (86%) described themselves as moderately active, while 14% reported being very active; none identified as athletes.

Body mass index (BMI) distribution was as follows: 3% underweight, 63% normal weight, 24% overweight, and 10% obese. When asked about body composition goals, 48% aimed to lose weight, 17% to maintain current weight, and 34% to increase muscle mass.

Self-reported dietary patterns and health conditions included: healthy eating (*n* = 905; 76.9%), Mediterranean diet (*n* = 673; 57.2%), lactose intolerance (*n* = 104; 8.8%), irritable bowel syndrome (*n* = 88; 7.5%), gluten-free diet (*n* = 68; 5.8%), fatty liver (*n* = 39; 3.3%), hypertension (*n* = 32; 2.7%), flexitarian diet (*n* = 29; 2.5%), ovo-lacto-vegetarian diet (*n* = 22; 1.9%), shellfish allergy (*n* = 22; 1.9%), and other conditions (*n* = 107; 9.1%).

### 3.3. Biochemical Blood Test Overall Results

**Figure 2:**
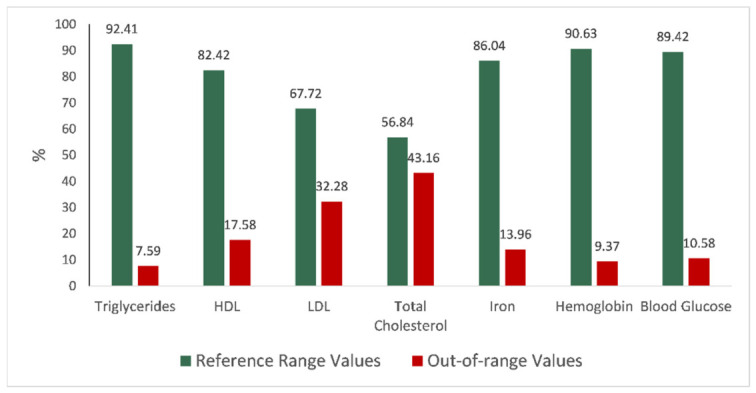
Distribution of blood test results expressed as the percentage of participants with values within the reference range versus those out of range for each parameter.

Analysis of the results for the baseline blood tests (Figure 2) showed that the majority of participants had values within the established reference ranges for most parameters. Iron (86.04%), hemoglobin (90.63%), blood glucose (89.42%), and triglycerides (92.41%) displayed the highest proportions of in-range results. In contrast, total cholesterol and LDL cholesterol showed substantially higher proportions of out-of-range values (43.16% and 32.28%, respectively), suggesting a notable prevalence of dyslipidemia in the assessed population. Triglycerides are strongly influenced by short-term factors such as recent dietary intake, fasting status, and acute metabolic fluctuations, and therefore often remain normal unless individuals present with metabolic syndrome or uncontrolled diabetes [36]. Conversely, total cholesterol (driven largely by its LDL component) reflects longer-term influences, including habitual dietary patterns, age-related lipid changes, and genetic predispositions affecting lipid metabolism. A personalized nutrigenetic test can motivate the participant to follow an appropriately tailored diet in order to improve total cholesterol levels by targeting the specific genetic and metabolic pathways involved in lipid regulation.

### 3.4. Nutrigenetics Overall Results

**Figure 3:**
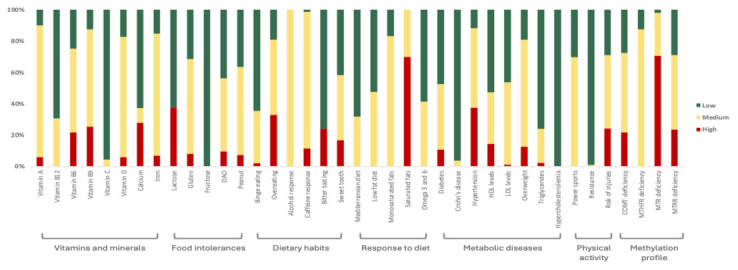
Frequency distribution of risk levels across the nutritional categories analyzed in the nutrigenetics report for the 357 participants.

The genotypes for 300 variants in 357 participants (91 men, 267 women; Supplementary Table S2 in File S3) were used to generate a genetic report for 39 features associated with nutritional aspects (Figure 3). The report provided specific nutritional recommendations according to the risk obtained from the analysis of the genetic variants associated with each feature.

From the 300 polymorphisms analyzed for the 39 nutritional features, only two genetic variants presented a significant difference (adjusted *p*-value < 0.05; Supplementary Table S3 in File S3) in frequency compared to the Iberian population from the 1000 Genomes project [37]. These polymorphisms are associated with two nutritional features, overweight and LDL levels, in the Metabolic diseases category.

Variants associated with Vitamin D deficiency, iron deficiency, diamine oxidase (DAO) intolerance, and methylenetetrahydrofolate reductase (*MTHFR*) deficiency were present in over 50% of the cohort. Also, as expected from previous indications [38], the frequency of the derived allele for rs4988235 variant (old nomenclature 13,910*T) in the *MCM6/LCT* region, associated with the lactase persistence trace, is considerably lower than in the North of Europe, so that 40% of the Spanish cohort is lactose intolerant and prone to symptoms such as bloating, gas, abdominal pain, stomach rumbling, and diarrhea when ingesting lactose.

### 3.5. Gut Microbiome Overall Results

The relative abundance of gut bacteria (Supplementary Table S4 in File S3) for the 200 participants (first batch) allowed us to generate an individual report with 32 features on gut microbiome features and nutritional suggestions to improve specific features when quantitative values were statistically distinct from those of a reference population. For example, participants who had an excess of lipid-degrading bacteria compared with the reference population (value on the >97.5% interval of the normal distribution) received the indication to reduce overall fat intake and, preferably, to replace animal fat with fish fat.

**Figure 4:**
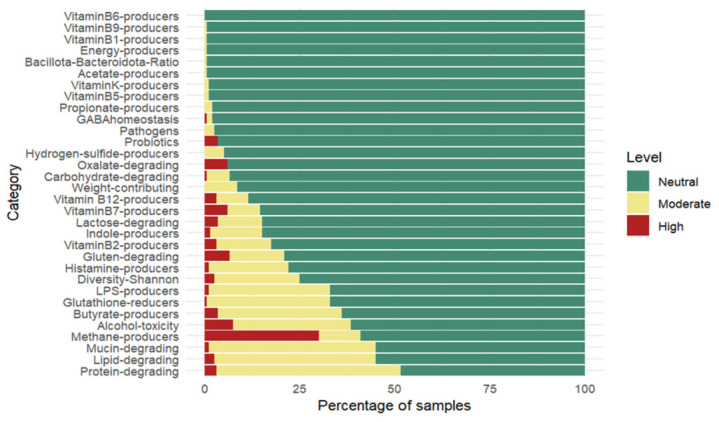
Ranked distribution of the proportions of the cohort displaying microbial functional dysregulations (Moderate and High) and reference levels (Neutral) across the analyzed categories.

Overall, the ranking analysis of microbiome features revealed that a proportion of individuals exhibited moderate-to-high dysregulation in some features (Figure 4). The most frequently altered groups were protein-degrading bacteria (51.5%; 95% CI: 44.6–58.4%), lipid-degrading bacteria (45.0%; 95% CI: 38.1–51.9%), mucin-degrading bacteria (45.0%; 95% CI: 38.1–51.9%), and methane producers (41.0%; 95% CI: 34.2–47.8%), which showed the highest prevalence of dysregulation in the studied cohort. Additional features, including bacteria associated with alcohol toxicity (38.5%; 95% CI: 31.8–45.2%), butyrate production (36.0%; 95% CI: 29.3–42.7%), lipopolysaccharide (LPS) production (33.0%; 95% CI: 29.3–42.7%), and glutathione reduction (33.0%; 95% CI: 29.3–42.7%), also displayed altered frequencies above 30%. In contrast, microbial categories related to vitamin biosynthesis (B5, K, B1, and B9) were consistently maintained within reference levels, with dysregulation detected in 1% or less of the individuals. Similarly, acetate producers, energy producers, and the Bacillota-to-Bacteroidota ratio exhibited minimal alterations, being moderately dysregulated in only one participant.

**Figure 5:**
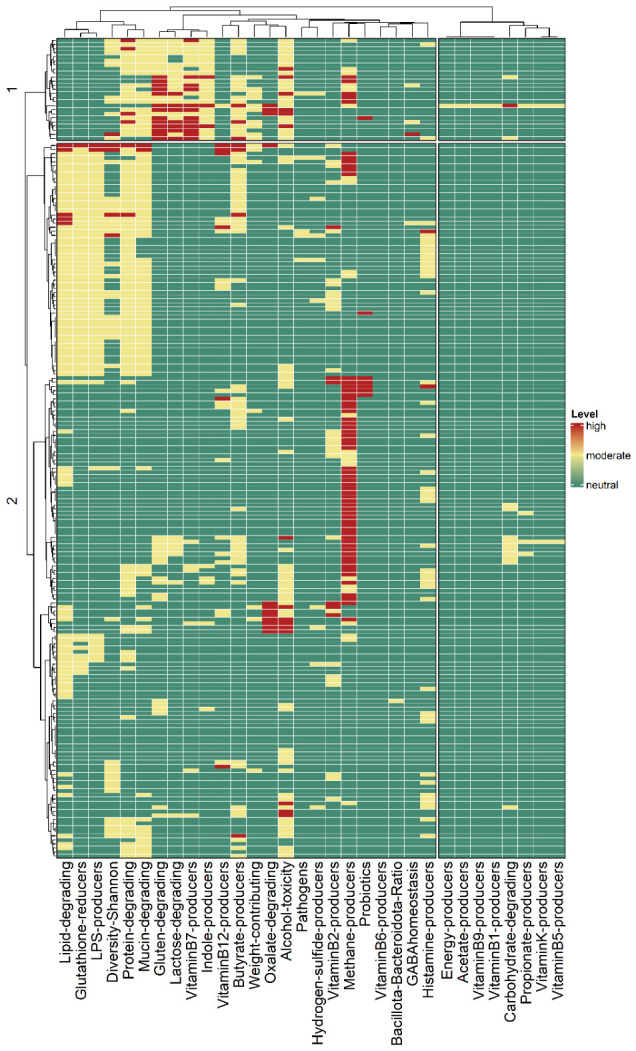
Heatmap of microbial functional populations across samples. Hierarchical clustering using Ward.D2 linkage and Euclidean distance separated the samples (lines) and categories (columns) into two distinct clusters. Green corresponds to reference levels for the specific microbiome feature, while yellow and red indicate moderate and high dysregulated levels. The numbers 1 and 2 indicate the two main clusters.

To visualize the global distribution of microbial functional categories, a heatmap was constructed with hierarchical clustering of samples based on their functional profiles (Figure 5). Clustering was performed using the Ward.D2 method with Euclidean distance, which minimizes within-cluster variance while maximizing between-cluster separation. This approach identified two distinct clusters of samples and two major groups of microbial functions, highlighting functional heterogeneity across individuals. The statistical validity of these clusters was confirmed by PERMANOVA, which demonstrated a significant separation between the two groups (F = 177.02, R^2^ = 0.47, *p* = 0.001). Further, feature-level pairwise Wilcoxon rank-sum tests (Supplementary Table S5 in File S3) identified 17 microbial categories significantly contributing to cluster differentiation (FDR-adjusted *p* < 0.05, Benjamini–Hochberg correction). Among these, indole producers, vitamin B7 producers, and lactose- and gluten-degrading bacteria were strongly enriched in one of the clusters (FDR-adjusted *p* < 1 × 10^−14^).

### 3.6. Analysis of the Personalized Nutrition Experience

Across all participants, the mean session time was 7.07 min, defined as the duration of a user’s interaction with the digital nutrition experience during a single visit, including time spent navigating the platform, exploring products, reviewing personalized recommendations, and receiving real-time guidance throughout the shopping journey. The longest engagement was observed in the recipe and nutritional planning modules. Compared to baseline metrics from January to April, use of the e-commerce platform increased by 154% during the experiment.

Adherence to nutritional recommendations was assessed through 912 completed feedback questionnaires. Among respondents, 35% reported following 50–75% of the recommendations, 31% followed 25–50%, 29% followed 0–25%, and 5% followed 75–100%. In total, 71% of participants followed at least 25% of the proposed recommendations.

The main reasons reported for non-adherence were: personal issues (34%), other unspecified reasons (24%), lack of palatability (10%), gastrointestinal symptoms (10%), lack of motivation (10%), high food prices (6%), and illness (4%).

The platform engagement analysis showed that 28% of participants created a personalized nutrition plan, while 72% did not. A total of 25 accounts were deleted during the study period.

### 3.7. Quantitative Analysis of Changes in Food Consumption Behavior—Analysis of the Second Microbiome Sample After 1 Month

We conducted a second microbiome analysis, with collection of the second stool sample around 1 month after the participants received the microbiome report. A total of 97 individuals (out of 200) agreed to be included in this second batch (Supplementary Table S6 in File S3). Changes in the measures of microbiome diversity are a quantitative indicator of the impact of behavioral changes on food consumption.

**Figure 6:**
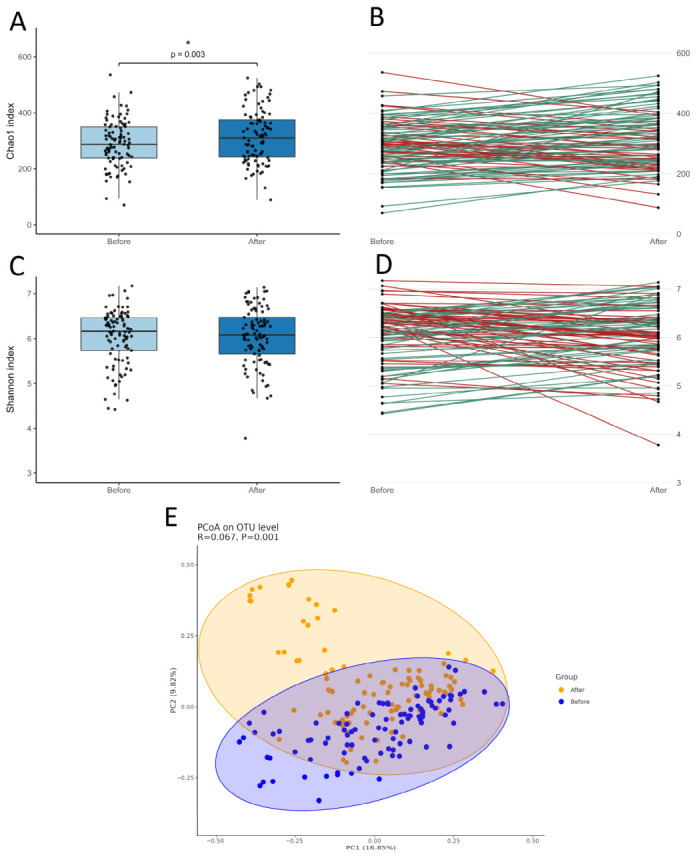
Changes in microbiome diversity measures in individuals (*n* = 97) that participated in the two collection batches (second stool collection approximately 1 month after participants received the first microbiome report). (**A**,**C**) Box plots for Chao1 and Shannon measures (* indicates significant *p*-values); (**B**,**D**) plots representing the changes in Chao1 and Shannon individual values (red indicates decrease and green indicates increase). (**E**) Principal Coordinates Analysis (PCoA) for the Bray–Curtis distances in community composition between before (blue) and after (orange) batches.

We focused on three measures of microbiome diversity. The widely used Shannon diversity takes into account richness (number) and evenness (abundance) of the bacterial species that constitute the microbiome. The Chao1 diversity only accounts for the number of bacterial species in the microbiome. Theoretically, changes will be faster for Chao1 than for Shannon. Our cohort presented a significant increase in microbiome diversity for the Chao1 measures, with 66% of the individuals showing higher values on the second batch (*n* = 97; Before, 286.059 bacterial species; After, 308.769; *p*-value = 0.003; Figure 6A,B). Differences were not statistically significant for the Shannon measure (50.5% showed an increase; Before, 6.166; After, 6.08; *p*-value = 0.4919; Figure 6C,D). The Bray–Curtis distances revealed significant differences in community composition between before/after batches, as confirmed by PERMANOVA (pseudo-F = 13.69, *p* = 0.001; Figure 6E). This indicates that the centroids of the batches—that is, their overall community structures—are significantly different. Importantly, the PERMDISP test was not significant (*p* = 0.097; Supplementary Figure S5 in File S1), suggesting that the dispersion (i.e., within-batch variability) did not differ substantially between batches. Together, these results support the conclusion that the observed differences are driven by true shifts in community composition rather than differences in variability or spread among samples. This strengthens the interpretation that the batches are compositionally distinct, rather than one batch simply exhibiting greater heterogeneity.

Given the short period between microbiome batches, participants who adhered to the recommendations already showed improvements in the number of species, but the time was probably not sufficient to significantly impact the evenness of the microbiome.

## 4. Discussion

By organizing the GENIE digital platform into three modules, we have managed to render the system efficient and easy for users to navigate. Module “enriched product data” demonstrates the feasibility of converting large-scale retail product assortments into structured, health-relevant datasets, providing a robust foundation for personalized nutrition and informed consumer choices. Module “data center (user vault)” illustrates how sensitive health information can be securely operationalized to drive personalized nutrition. By integrating blood, urine, microbiota, and genetic biomarkers, the system reflects current scientific evidence on the role of multi-omic data in shaping individualized dietary strategies [8]. The approach provides a clinically relevant framework for translating emerging nutrigenetics and microbiome research into real-world interventions, aligning with growing literature on precision nutrition as a tool for chronic disease prevention and health optimization [8,9,12]. Module “Personalized user experience and supermarket integration” demonstrates the feasibility of translating diagnostic insights into actionable dietary interventions embedded within daily purchasing behavior.

The GENIE experience provided valuable insights into nutrition-related consumer attitudes and behavior that go beyond theoretical assumptions. The fact that respondent participants were 18% men and 82% women shows the strong sex bias in awareness and openness to new personalized tools. This sex bias is shared across the overall well-being market [39]. The observed sex bias in participation may also reflect sex differences in grocery shopping habits, as women are more often the primary household shopper [40]. The personalization engine enabled integration of individual health profiles with the supermarket product database. Across participants, mean session time was 7.07 min, with the highest engagement in recipe and nutrition planning modules. Use of the e-commerce platform increased by 154% compared to the January–April baseline, suggesting a strong engagement effect of the personalized nutrition intervention. Adherence to recommendations, assessed through 912 feedback questionnaires, showed that 71% of participants followed at least 25% of the suggestions, with main barriers including, in decreasing order, personal issues, lack of motivation, palatability, and cost. Platform engagement analysis showed that 28% of participants created a personalized nutrition plan, demonstrating that a subset of users actively engaged with advanced features. While encouraging, the majority (72%) did not create a plan, suggesting that factors such as usability, time constraints, or motivation may have limited broader adoption. This highlights opportunities to enhance user engagement and increase uptake of personalized planning tools.

Incorporating nutrigenetic information into dietary planning contributes to more personalized nutritional strategies aimed at mitigating deficiency-related health risks and promoting overall health, not only at the individual level but also at the population level. As we have seen for this Spanish cohort, even if the allele frequencies are typical of the population and compatible with normal physiology, the entire group is nevertheless predisposed to some nutritional limitations and could benefit from further caution on diet planning. Specifically, deficiencies in vitamin D, iron, and *MTHFR*, as well as *DAO* intolerance, reached a 50% frequency of the cohort, and lactose intolerance reached a 40% frequency. These features are explained by the Iberian frequencies of associated variants, such as rs12785878, rs1800562, and rs4988235 for vitamin D and iron deficiencies and lactose intolerance, respectively. These variants are known or candidates for natural selection, and show gradients of frequencies across Europe [41,42,43]. It is increasingly evident that Iberian nutritionists must be aware of the high abundance of these profiles in the population, and advise for a more generalized nutrigenetic testing, as simple dietary interventions can improve the well-being of the Iberians significantly.

The gut microbiome analyses showed that microbial categories related to vitamin biosynthesis (B1, B5, B9, and K) were consistently maintained within reference levels, likely reflecting the functional resilience of the gut microbiome and its role in maintaining host nutrient homeostasis. In contrast, the analyses revealed that the most frequently altered features were protein-, lipid-, mucin-degrading bacteria, and methane producers. Elevated proteolytic activity in the gut microbiome can result in the accumulation of metabolites such as ammonia, phenols, and indoles, many of which are known to increase pro-inflammatory responses in the intestinal epithelium, which may compromise gut barrier integrity and overall gut health [44]. Dysregulated mucin degradation can also compromise the mucus barrier [45], allowing closer microbial contact with the host epithelium and potentially triggering inflammation or barrier dysfunction. This shift could reflect dietary imbalances, microbial dysbiosis, or other host-related factors, highlighting the importance of monitoring proteolytic activity and mucin-degrading bacteria in personalized nutrition interventions. Gut bacteria involved in lipid metabolism play a key role in degrading dietary fats and regulating host lipid homeostasis. Imbalances in microbial lipid metabolism can disrupt bile acid composition, promote the production of pro-inflammatory metabolites, and contribute to metabolic disturbances such as dyslipidemia and low-grade inflammation [46]. Alterations in the abundance of lipid-degrading bacteria have been linked to high-fat diets and obesity-associated microbiome shifts [47], underscoring the importance of maintaining balanced microbial lipid metabolism for both gut and systemic metabolic health. Similarly, an increased abundance of methane producers, which utilize fermentation byproducts such as hydrogen generated during lipid and other substrate metabolism, may reflect altered microbial fermentation dynamics associated with high-fat diets [48]. Collectively, these results indicate that the most dysregulated microbial functions—protein, lipid, and mucin degradation, along with methanogenesis—reflect a shift toward a metabolically imbalanced gut ecosystem. In terms of the two groups in the overall analysis, the group with the four most dysregulated functions (imbalances in proteolytic metabolism, such as indole, gluten; micronutrient biosynthesis of vitamin B7; and carbohydrate fermentation of lactose) suggests a microbiome configuration that may contribute to intestinal inflammation, immune activation, and impaired nutrient utilization. In this study, we observed that a relatively short intervention period of approximately one month following personalized recommendations was associated with an increase in microbiome diversity in about 70% of participants (Bray–Curtis distances were statistically significant; *p* = 0.001). These findings suggest that even short-term nutritional interventions may have the potential to positively influence aspects of gut microbial composition.

This study has certain limitations. It is a single-arm study, so no control group was included. As it was a participatory study, the selection of participants could not be rigorously controlled (restricting, for example, the adjustment for confounders), and the participants’ feedback was necessarily an important output. The funding agency did not interfere with the study, but imposed a one-year timeline, which was also an important limiting factor. For that reason, microbiome change and engagement metrics were used as proxies, instead of other dietary measures that would need a longer testing period, such as a validated nutrition-quality index from purchased foods before, during, and after the intervention. The level of interaction with the three groups throughout the project was heterogeneous, being longer with Group 3 than with Group 1, which did not allow for more complete comparisons between groups. Overall, these limitations imply that the obtained results should be interpreted with caution. Nevertheless, this study supports the potential value of incorporating multi-omics personalized strategies into routine shopping decisions.

## 5. Conclusions

By coupling nutrient gap analysis with real-world food environments, the GENIE approach provides a scalable framework for advancing precision well-being within retail ecosystems.

## Data Availability

All data supporting the findings of this study are available within the Supplementary Materials.

## Supplementary Materials

The following supporting information can be downloaded at: https://www.mdpi.com/article/10.3390/nu18101528/s1, File S1: Supplementary Figures S1–S5. File S2: Questionnaires. File S3: Supplementary Tables S1–S6.

## Abbreviations

The following abbreviations are used in this manuscript:

GENIE	Genomic Evaluation and Nutritional Integration Experience
DNA	Deoxyribonucleic Acid
rRNA	Ribosomal ribonucleic Acid
SNP	Single Nucleotide Polymorphisms
FTO	Fat-mass and obesity-associated gene
BMI	Body mass index
LDL-cholesterol	Low-Density Lipoprotein cholesterol
PN	Personalized Nutrition
PREDICT	Personalised Responses to Dietary Composition
FeFiFo Study	Fermented and Fiber-rich Food Study
DTC	Direct-To-Consumer
EU	European Union
GDPR	General Data Protection Regulation
APIs	Application Programming Interface
AI	Artificial intelligence
PDF	Portable Document Format
REST API	Representational State Transfer Architectural Interface
LOPDGDD	Protection of Personal Data and guarantee of digital rights
DQC	Data Quality Control
VCF	Variant Call Format
DADA2	Divisive Amplicon Denoising Algorithm 2
PERMANOVA	Permutational Multivariate Analysis of Variance
FDR	False Discovery Rate
CI	Confidence Intervals
DAO	Diamine Oxidase
MTHFR	Methylenetetrahydrofolate Reductase
LPS	Lipopolysaccharide
ASVs	Amplicon Sequence Variants
