# Supplementary material for: An Experiment in Personalized Shopping for Optimal Health, with Integration of Nutrigenetics and Gut Microbiome Information
Source: Nutrients. 2026 May 12;18(10):1528. doi: 10.3390/nu18101528 (PMC13209603; doi:10.3390/nu18101528)
Supplement: Supplementary file 1 [file nutrients-18-01528-s001.zip › FileS1.pdf]

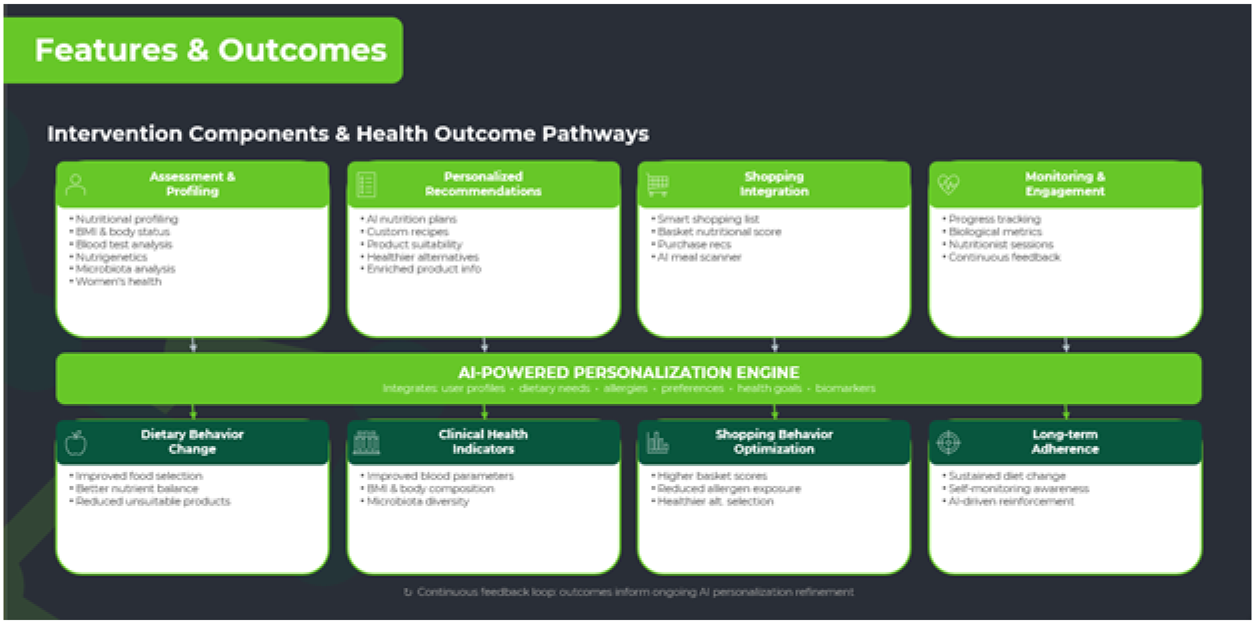

Supplementary Figure S1. GENIE digital platform, features and outcomes. The platform generates personalized nutrition recommendations and delivers them through connected digital tools. In panel, an API transfers personalized shopping lists to the retailer's ecosystem, allowing users to access and manage them directly via the retailer's app or e-commerce platform, supporting convenient and practical use.

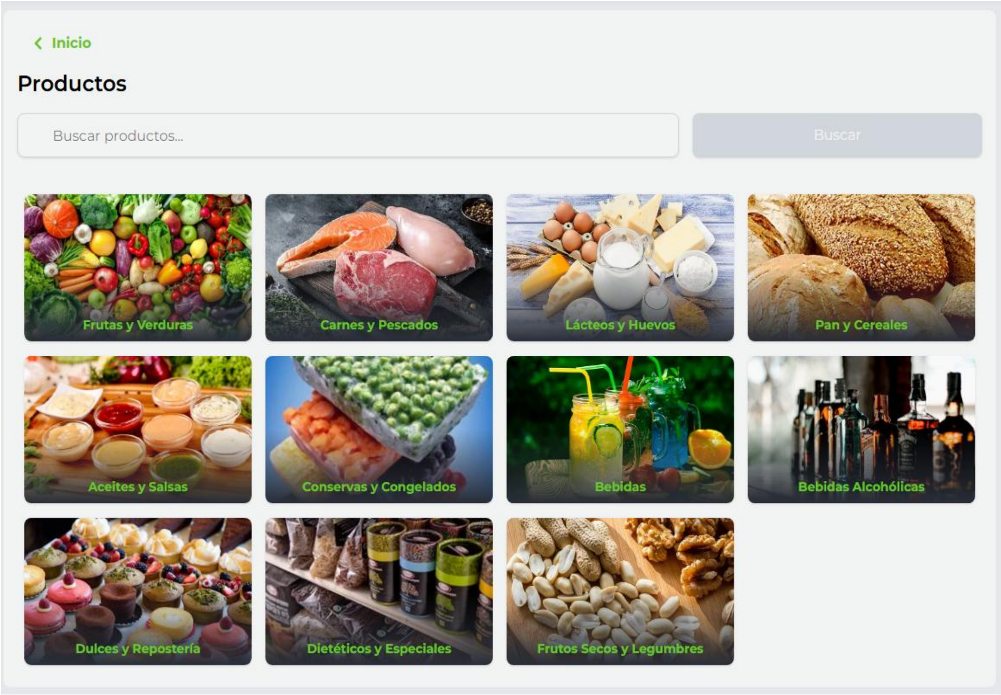

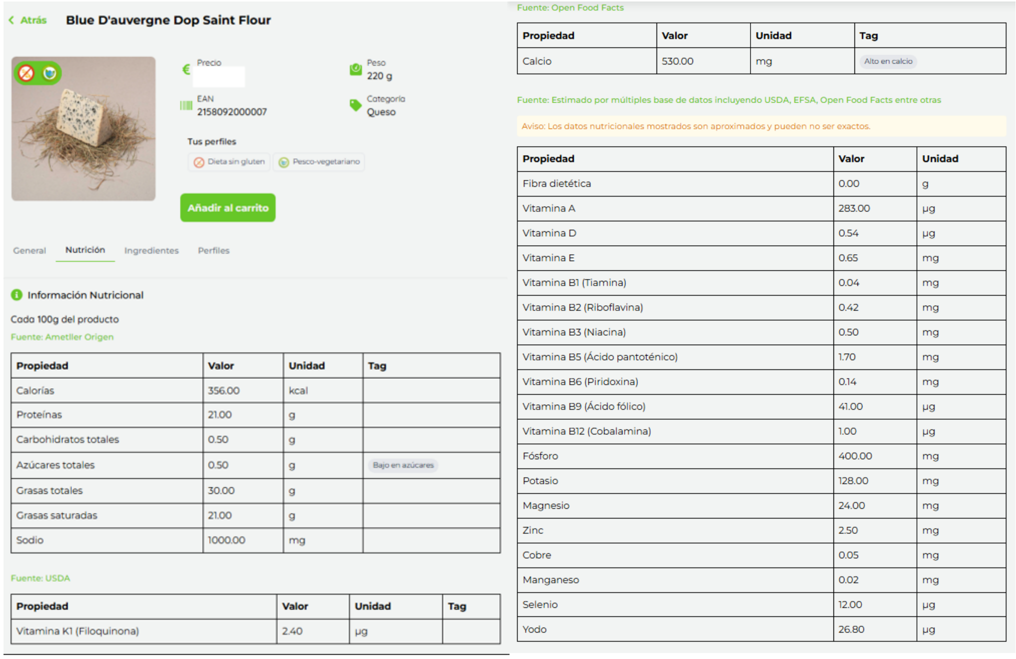

Propiedad	Valor	Unidad	Tag
Calorías	356.00	kcal	
Proteínas	21.00	g	
Carbohidratos totales	0.50	g	
Azúcares totales	0.50	g	Bajo en azúcares
Grasas totales	30.00	g	
Grasas saturadas	21.00	g	
Sodio	1000.00	mg	

Fuente: USDA

Propiedad	Valor	Unidad	Tag
Vitamina K1 (Filoquinona)	2.40	µg	

Fuente: Open Food Facts

Propiedad	Valor	Unidad	Tag
Calcio	530.00	mg	Alto en calcio

Fuente: Estimado por múltiples base de datos incluyendo USDA, EFSA, Open Food Facts entre otras

Aviso: Los datos nutricionales mostrados son aproximados y pueden no ser exactos.

Propiedad	Valor	Unidad
Fibra dietética	0.00	g
Vitamina A	283.00	µg
Vitamina D	0.54	µg
Vitamina E	0.65	mg
Vitamina B1 (Tiamina)	0.04	mg
Vitamina B2 (Riboflavina)	0.42	mg
Vitamina B3 (Niacina)	0.50	mg
Vitamina B5 (Ácido pantoténico)	1.70	mg
Vitamina B6 (Piridoxina)	0.14	mg
Vitamina B9 (Ácido fólico)	41.00	µg
Vitamina B12 (Cobalamina)	1.00	µg
Fósforo	400.00	mg
Potasio	128.00	mg
Magnesio	24.00	mg
Zinc	2.50	mg
Cobre	0.05	mg
Manganeso	0.02	mg
Selenio	12.00	µg
Yodo	26.80	µg

Supplementary Figure S2 – GENIE digital platform print-screen of the module enriched product data.

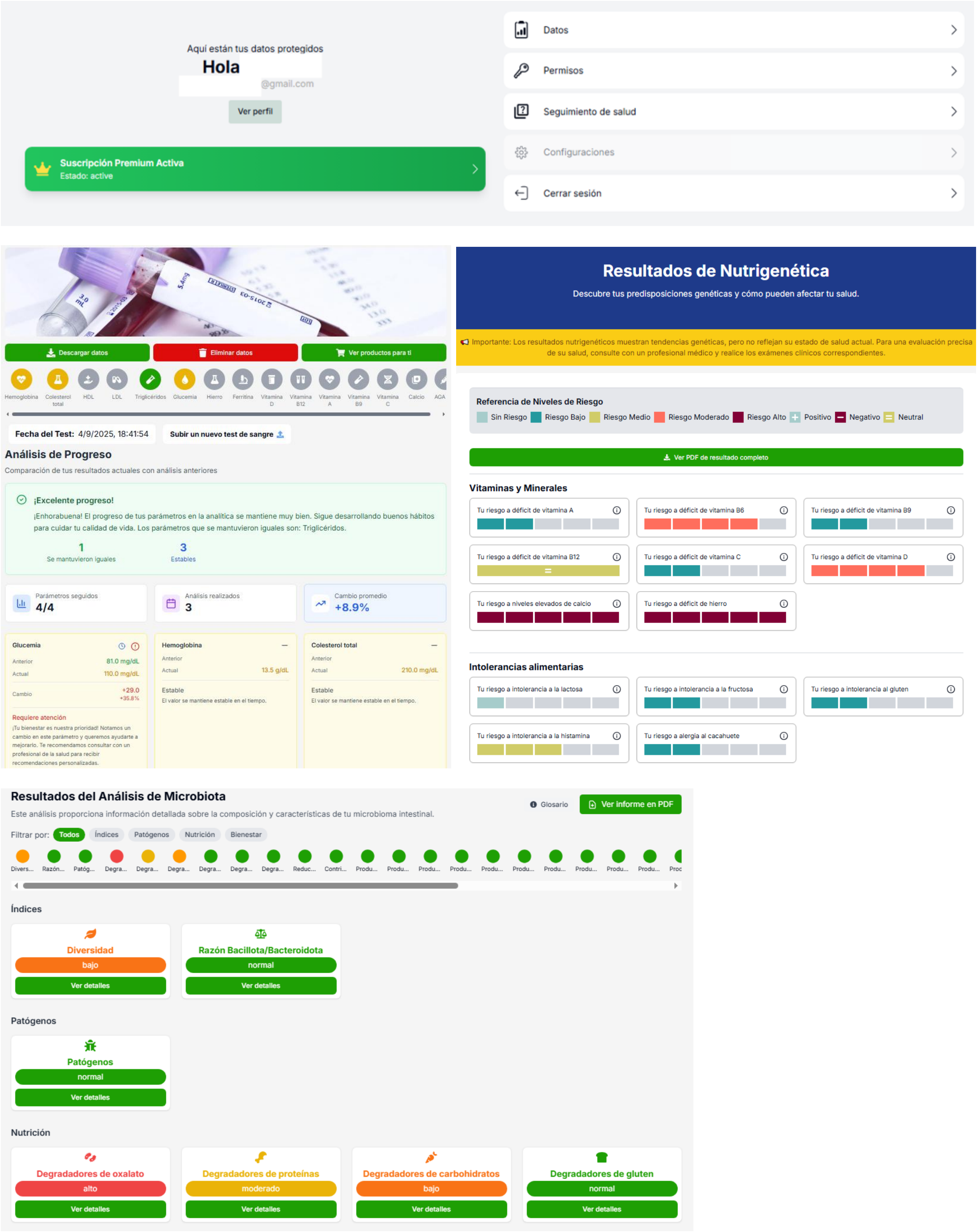

Supplementary Figure S3 – GENIE digital platform print-screen of the module data center (user vault).

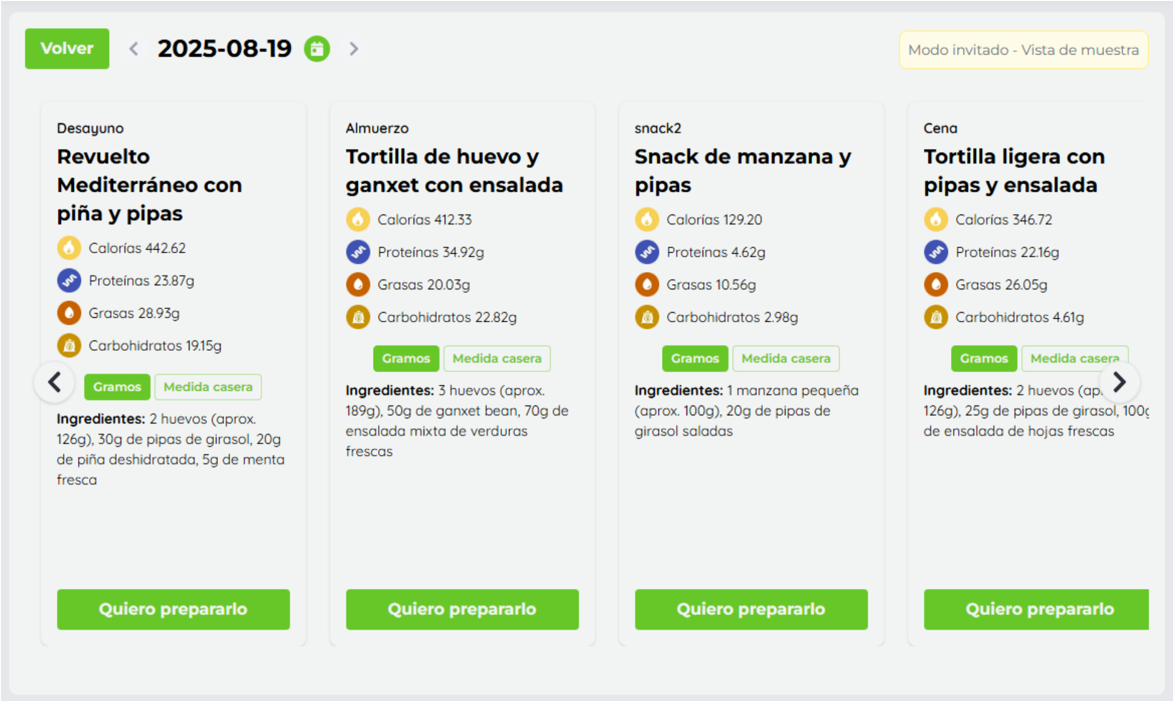

Supplementary Figure S4 – GENIE digital platform print-screen of the module personalized user experience and supermarket integration.

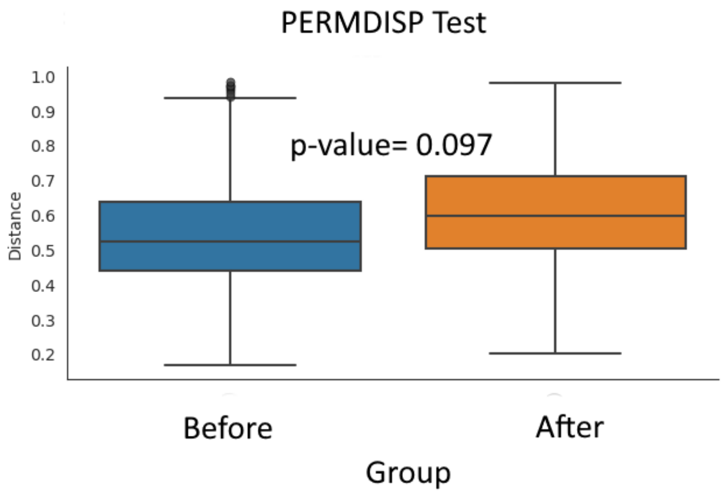

Supplementary Figure S5 - PERMDISP tests between the two collection batches (second stool collection near 1 month after participants received the first microbiome report).
