# Supplementary material for: An Experiment in Personalized Shopping for Optimal Health, with Integration of Nutrigenetics and Gut Microbiome Information
Source: Nutrients. 2026 May 12;18(10):1528. doi: 10.3390/nu18101528 (PMC13209603; doi:10.3390/nu18101528)
Supplement: Supplementary file 1 [file nutrients-18-01528-s001.zip › FileS2.pdf]

Questionnaire 1 — Personalized Nutrition Data Intake Form

Step 1 of 5 — Personal Information

Basic physical data

- **Biological Sex**
  - Male
  - Female
- **First Name**
- **Last Name**
- **Weight** (kg)
- **Height** (cm)
- **Date of Birth** (DD-MM-YYYY)
- **Waist Circumference** (cm) *(Optional)*

Step 2 of 5 — Lifestyle

Daily habits and goals

- **Occupation**
(Select your daily activity level)
- **Meals per Day**
  - Breakfast
  - Mid-morning snack

- Lunch
- Afternoon snack
- Dinner

- **Body Composition Goal**
  - Lose weight
  - Maintain current state
  - Increase muscle mass

Step 3 of 5 — Physical Activity

Sports and exercise routine
Up to 3 activities

For each activity:

- **Type of activity** (e.g., Strength training, Running, Swimming, etc.)
- **Days of the week**
  - Mon / Tue / Wed / Thu / Fri / Sat / Sun
- **Hours of exercise per day**
  - 30 minutes
  - 1 hour
  - 1.5 hours
  - 2+ hours

(Example)
Resistance Training (weightlifting, strength training, vigorous effort)

- Days: Mon, Thu, Sat
- Duration: 1 hour/day

Running (general)

- Days: Tue, Fri
- Duration: 30 minutes/day

Step 4 of 5 — Nutritional Profile

Choose your dietary profiles *(Select up to two)*

- Healthy eating
- Vegetarian
- Vegan
- Celiac / Gluten-free
- Diabetes Type 1 or Type 2
- Pregnancy
- Fatty liver
- Hypertension
- Irritable bowel syndrome
- Mediterranean diet

Step 5 of 5 — Allergies & Intolerances

Food restrictions and sensitivities

- **Add Allergy +**
- **Add Intolerance +**

Selected Allergies:

- Soy allergy *(example)*

Selected Intolerances:

- Gluten intolerance *(example)*

- **Advanced Screening Questionnaire (Level 2).** Administered to participants fulfilling eligibility criteria and opting for deeper personalization. This survey collected detailed clinical history (e.g., chronic conditions, digestive disorders), psychological and behavioral traits related to eating (e.g., emotional eating, binge tendencies), vitamin and mineral intake sources, adherence to dietary patterns (e.g., Mediterranean diet), and previous responses to dietary interventions.

Questionnaire 2 — Clinical history, psychological and behavioral data related to eating

Question	Options
**Eating habits and diets:**	
Do you follow any specialized diet (paleo, halal, kosher, low-FODMAP, raw vegan, keto, low-grain, etc.)?	"1. YES > Which one? 2. NO"
Do you exclude certain foods from your diet, such as dairy, nightshades (eggplant, tomato, potato) or products with refined sugar?	"1. YES > Which ones? 2. NO"
How often do you consume unprocessed raw foods?	"1. Never 2. 1–3 times per week 3. 4–5 times per week 4. Almost every day"
Do you have any dietary restriction not mentioned in the categories above?	"1. YES > Which one? 2. NO"
How often do you consume foods prepared at home?	"1. Never 2. 1–3 times per week 3. 4–5 times per week 4. Almost every day"
**Specific food consumption:**	

How often do you consume fresh vegetables and fruits? (includes raw, cooked, and frozen)	"1. Never 2. 1–3 times per week 3. 4–5 times per week 4. Almost every day"
How many units of fresh vegetables and fruits do you consume per day?	"1. Less than 1 unit 2. 2–4 units per day 3. 5 or more units per day"
How often does the participant consume the following animal-based foods: beef, pork, turkey, chicken, eggs?	"1. Never 2. 1–3 times per week 3. 4–5 times per week 4. Almost every day"
How often does the participant consume plant-based foods such as plant-based milk (soy, almond, rice), tofu, whole grains like legumes, quinoa?	"1. Never 2. 1–3 times per week 3. 4–5 times per week 4. Almost every day"
How often do you consume fermented products such as Greek yogurt, kefir, kombucha, sauerkraut, or kimchi?	"1. Never 2. 1–3 times per week 3. 4–5 times per week 4. Almost every day"
How often do you consume products such as bread, pasta, oats, or rice?	"1. Never 2. 1–3 times per week 3. 4–5 times per week 4. Almost every day"
How often do you consume fish or seafood?	"1. Never 2. 1–3 times per week 3. 4–5 times per week"
How often do you consume salty snacks, sweets, or foods with refined sugar?	"1. Never 2. 1–3 times per week 3. 4–5 times per week 4. Almost every day"
How often do you consume sugary drinks such as sodas or juices?	"1. Never 2. 1–3 times per week 3. 4–5 times per week 4. Almost every day"

How often do you consume olive oil?	"1. Never 2. 1–3 times per week 3. 4–5 times per week 4. Almost every day"
How often do you drink water?	"1. Less than 1 liter/day 2. 1–1.5 liters/day 3. 1.5–2 liters/day 4. More than 2 liters/day"
What is your main source of water?	"1. Bottled 2. Tap"
**Supplements, medications, personal care products:**	
Do you take any nutritional supplement (multivitamin, omega-3, vitamin B, vitamin D, probiotics, others)?	"1. YES > Which ones? > 1. Multivitamin 2. B-complex vitamins 3. Vitamin D 4. Prebiotics 5. Probiotics 6. Omega-3 7. Others: which?"
How often do you brush your teeth?	"1. Always after eating 2. Once a day 3. Only twice a day 4. I don't brush my teeth"
Do you take contraceptive pills?	"1. YES 2. NO"
**Lifestyle habits:**	
Do you smoke tobacco or cannabis?	"1. YES > Which one? 2. NO"
How often do you consume alcohol?	"1. Never 2. 1–3 times per week 3. 4–5 times per week 4. Almost every day"
Do you use a swimming pool or jacuzzi?	"1. YES 2. NO"

How many hours do you sleep on an average night?	"1. Less than 5 hours/day 2. 6–7 hours/day 3. 7–8 hours/day 4. 8–9 hours/day 5. More than 9 hours/day"
Do you have any sleep-related condition (insomnia, sleep apnea)?	"1. YES > Which one? 2. NO"
**Diagnoses and health conditions:**	
Have you been diagnosed with any disease (IBD, IBS, lung, intestinal, liver, kidney disease, migraines, skin diseases, seasonal allergies, etc.)?	"1. YES > Which one? 2. NO"
Have you been diagnosed with diabetes, thyroid disease, or autoimmune diseases?	"1. YES > Which one? 2. NO"
Have you been diagnosed with any mental condition such as depression, anxiety, bipolar disorder, schizophrenia, etc.?	"1. YES > Which one? 2. NO"
Have you been diagnosed with any psychological or mental health condition in the last year?	"1. YES > Which one? 2. NO"
Do you have any skin condition such as psoriasis or dermatitis?	"1. YES > Which one? 2. NO"
Have you been diagnosed with any type of allergy/intolerance, food-related or not?	"1. YES > Which one? 2. NO"

Do you have seasonal allergies or respiratory problems such as asthma?	"1. YES > Which one? 2. NO"
Do you suffer from migraines or frequent headaches?	"1. YES 2. NO"
Have you or a family member had cancer of the esophagus, stomach, pancreas, small intestine, colon, rectum, anus, or liver?	"1. YES > Who/which? 2. NO"
Have you had or do you currently have infectious gastritis diagnosed as Helicobacter pylori?	"1. YES > Did you take antibiotics for gastritis? 2. NO"

Body weight changes:

Have you had a significant change in your weight or health status in the last year? (more or less than 10% of your initial total weight)	"1. YES > Weight gain or loss? 2. NO"

Surgeries and medical procedures:

Have you had an appendectomy?	"1. YES 2. NO"
Have you undergone any surgery (tonsils, removed organs)?	"1. YES 2. NO"
Have you undergone bariatric surgery or any weight-related surgery?	"1. YES > Which one? 2. NO"

Other factors:

Were you born through natural childbirth or C-section?	"1. Natural birth 2. C-section"
What type of environment do you live in?	"1. Urban 2. Rural"
Do you have roommates or live with other non-related individuals?	"1. YES > Do the people you live with also provide samples for the study? 2. NO"
Do you have pets (dogs, cats, birds, or others)?	"1. YES 2. NO"

- **Post-intervention Feedback Survey.** Offered after participants received personalized reports. This optional survey assessed comprehension of results, perceived usefulness of recommendations, satisfaction with the process, and anticipated impact on future dietary behaviors. Data was used to evaluate user experience and the perceived value of the GENIE solution.

Questionnaire 3 — GENIE Experience Feedback

GENIE Experience: Tell us what you thought!
Thank you for sharing your opinion. Your responses help us improve your experience.

---

1. How useful did you find the food recommendations based on your results? *

(1 = Not useful at all, 5 = Extremely useful)
Select

2. How would you describe the ease of use of the e-commerce platform to make your purchases? *

(1 = Very difficult, 5 = Super easy)
Select

3. Did you feel more confident choosing foods that match your results, nutritional profile, and nutritional needs? *

(1 = Not at all, 5 = Much more confident)
Select

4. How would you rate your overall experience on the platform? *

(1 = Poor, 5 = Excellent)
Select

5. Did you notice any positive changes in your nutrition, health, or well-being after the experience? *

- Yes, I noticed positive changes
- No, not yet

6. If you could improve one aspect of the experience, what would it be? (optional)

Open response

7. Would you recommend this service to a friend or family member? *

(1 = No, 5 = Absolutely)
Select

8. Would you pay for this experience in the future? *

(1 = No, 5 = Absolutely)
Select

9. What did you like the most about the experience? *

Open response

Post-experience Adjustment Survey. Offered to participants whose gut microbiota did not show the expected improvement after the GENIE program. This adaptive survey investigates potential behavioral, emotional, and lifestyle factors that may have influenced outcomes, including adherence to recommendations, dietary tolerance, stress, and routine changes. The data is used to understand barriers to progress and to refine personalized recommendations for next experiences.
